# Blockade of glutamate receptor ameliorates lipopolysaccharide-induced sepsis through regulation of neuropeptides

**DOI:** 10.1042/BSR20171629

**Published:** 2018-05-08

**Authors:** Zhai Zhe, Bi Hongyuan, Qiao Wenjuan, Wang Peng, Liu Xiaowei, Gao Yan

**Affiliations:** Department of Intensive Care Unit, The Fourth Affiliated Hospital of Harbin Medical University, Harbin 150001, China

**Keywords:** acute lung injury, glutamate, NMDAR, SP, VIP

## Abstract

Glutamate receptors (N-methyl-d-aspartate receptor (NMDAR)) are expressed mainly in the central nervous system (CNS), but several potentially important exceptions are worth mentioning. Recently, NMDAR, a glutamate receptor, has been reported to be found in the lungs. NMDAR is activated in acute lung injury (ALI). Here, the present experiment was designed to examine whether NMDAR blockade (MK-801) ameliorates ALI through affecting neuropeptides in LPS-induced sepsis animal models. Male Kunming mice were divided into control group, LPS group, control + MK-801 group, and LPS + MK-801 group. Bronchoalveolar lavage fluid (BALF) was collected and evaluated. The lung histological pathology was assayed by immunocytochemistry staining. Western blot was used to measure PGP9.5, substance P (SP), and vasoactive intestinal polypeptide (VIP). Results showed that LPS-induced mice animal models were ameliorated by co-treatment with the MK-801, an uncompetitive NMDAR antagonist. Moreover, the protective effects of MK-801 attributed to the increased secretion of VIP and decreased secretion of SP. The results of the present study indicated that the blockade of NMDAR may represent a promising therapeutic strategy for the treatment of sepsis-associated ALI through regulation of neuropeptides.

## Introduction

Glutamate (Glu) is the main excitatory neurotransmitter which acts on glutamate receptors in the central nervous system (CNS) but overactivation of these receptors can cause several damages to neural cells including death. Recent studies show that the glutamate agonist N-methyl-d-aspartate (NMDA) can trigger acute lung injury (ALI). ALI is a direct and indirect injury to alveolar epithelial cells and capillary endothelial cell, causing diffuse pulmonary interstitial and alveolar edema and acute hypoxic respiration failure. ALI is characterized by reduced lung volume and compliance, and imbalance of the ventilation/perfusion ratio, inducing hypoxemia and respiratory distress and its severe stage (oxygen index <200) known as acute respiratory distress syndrome (ARDS) [1].

Recent report showed that ALI induced by LPS has endogenous glutamate release, which was involved in ALI through the receptors [2,3]. Glutamate receptor blockers can effectively reduce the degree of ALI. Glutamate receptors are divided into two categories based on the mechanism by which their activation gives rise to a post-synaptic current: one for the ionotropic receptors, including: N-methyl-d-aspartate receptor (NMDAR), kainic acid receptor (KAR), and α-amino-3 hydroxy-5 methyl isoxazole-4 receptor (AMPAR), which are coupled with ion channel to form the receptor channel complex, mediating a fast signal transmission; the other belongs to a class of metabotropic receptors (mGluRs), which are coupled with membrane, produces a slow physiological response [4]. Numerous studies have shown that glutamate and its NMDAR play an important role in cell injury. NMDAR has been the focus of much attention because of its implication in cell injury and death in acute conditions [5]. MK-801 is a noncompetitive glutamate NMDA channel blocker which acts only on the activated receptor, not the receptor at rest, and has protective effects on hypoxia-induced lung cell injury, reducing the damage of alveolar capillary wall and the degree of pulmonary edema [6].

Vasoactive intestinal polypeptide (VIP) and substance P (SP) are neuropeptides widely distributed in the respiratory system of humans and animals. Many studies have found that VIP has significant anti-inflammatory and anti-injury effects [7] through inhibiting the release and adhesion of inflammatory cells [8] participating in the synthesis of enzyme protein against reactive oxygen species and has protective effects against injury. However, SP has significant proinflammatory effects and participates in inflammatory diseases of the respiratory, gastrointestinal, and musculoskeletal systems [9].

In the present study, we examined the expression of NMDAR in normal and sepsis lung tissues and assessed their possible role in lung protection by the use of selective blocker MK-801. Moreover, here, we reported the effects of MK-801 on the expression of VIP and SP in lungs which are involved in ALI in a variety of experimental models.

## Materials and methods

### Animals and grouping

Male Kunming mice (20–24 g) were provided by Animal Experimental Center of Harbin Medical University. The protocol was approved by the Ethics Committee Institute of the Fourth Affiliated Hospital of Harbin Medical University. Before the experiment, mice were placed in the laboratory for 1 week, and they were free to eat and drink. Mice (40) were divided into four groups: control group by injecting PBS (pH: 7.4) intraperitoneally; LPS group by injecting 30 mg.kg^−1^ LPS intraperitoneally; control + MK-801 group by injecting MK-801 (0.5 mg.kg^−1^) intraperitoneally; LPS + MK-801 group by injecting MK-801 (0.5 mg.kg^−1^) intraperitoneally before 30 min of LPS administration. The lung tissue injuries were determined by HE staining.

### Bronchoalveolar lavage fluid

The mice were given tracheal intubation after anesthesia. 1 ml of precold sterile PBS was slowly injected into the lung, and aspiration repeated three times. Lavage fluid was then collected and centrifuged at 1600 rpm at 4°C for 10 min. The protein content in the supernatant was measured by Lowry’s method. The red blood cells of the precipitating cells were destroyed by the red blood cell solution, and the blood cells were counted under the optical microscope. Glutamate Assay Kit was supplied by Sigma–Aldrich (Shanghai), China. This assay determines the concentration of glutamic acid by enzymatic reaction, and the reaction produces a colorimetric (450 nm) product proportional to the content of glutamic acid.

### Immunohistochemistry

To evaluate the distribution of neuropeptides, the lung histological pathology was done by using immunohistochemistry study. The right lungs were obtained and fixed in 4% paraformaldehyde, embedded in paraffin and cut into 5-μm sections. After endogenous peroxidase was blocked by 3% H_2_O_2_ for 20 min, the lung sections were incubated with rabbit anti-PGP9.5, VIP or SP polyclonal antibodies (Santa Cruz Biotechnology, 1:200 dilution) overnight at 4°C. Then the slides were incubated with biotinylated goat anti-rabbit IgG at 37°C for 1 h. After washing in PBS, the signal was detected with 3,3′-diaminobenzidine.

### Western blot

Protein was extracted from lung tissues and quantitative analysis was done by BCA method. After boiling the protein in sample buffer for 5 min, 50 μg protein was added in 10% polyacrylamide gel and electrophoresis was done at 120, 28 V for 2 h, and then transferred on to PVDF membrane under the condition of 100 A for 3 h. After 5% skim milk pretreatment, rabbit anti-NMDAR, PGP9.5, VIP, or SP polyclonal antibodies were added overnight at 4°C. Then the slides were incubated with biotinylated goat anti-rabbit IgG at 37°C for 1 h and assayed by ECL and X-ray imaging. The gray value of each band is measured using GAPDH as internal control.

### Real-time PCR

RNAs were isolated from lungs using TRIzol reagent (Invitrogen, U.S.A.) according to the instructions of the manufacturer. Each sample was reverse transcribed into cDNA and analyzed by quantitative real-time PCR. Quantitative real-time PCR was performed on ‘Applied Bio Systems Inc. 1900 system’ using the SYBR Green Real Time PCR Kit (Bio–Rad, U.S.A.). Each cycle included 94°C for 30 s, 60°C for 30 s after an initial 94°C for 4 min. Then, calculated the Δ*C*_t_ (average *C*_t_ − average *C*_t_ of GAPDH) and ΔΔ*C*_t_ (experimental Δ*C*_t_ − control Δ*C*_t_ ). Calculated the fold change from experimental to control as 2^−ΔΔ*C*_t_^ [10].

### Statistics

SPSS13.0 statistical software was used to analyze all data expressed by mean ± S.D. The comparison between groups was analyzed using a single-factor ANOVA (one-way ANOVA). The relationships between NMDAR and neuropeptides were explored by simple correlation and multiple regression analyses. *P*-value of less than 0.05 was considered significant.

## Results

### MK-801 ameliorated sepsis

**Figure 1:**
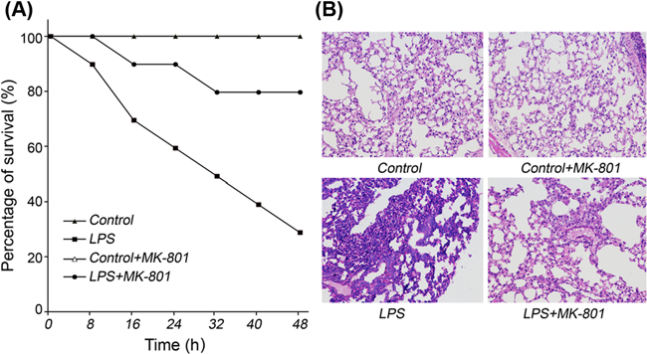
Protective effects of MK-801 on LPS-induced mice (*n*=10) Male Kunming mice were intraperitoneally administered LPS (30 mg.kg^−^^1^). MK-801 (0.5 mg.kg^−1^) was intraperitoneally injected before 30 min of LPS administration. (**A**) Survival rates were evaluated within 48 h after LPS administration. (**B**) Lung pathology was examined by HE staining. Pretreatment with MK-801 significantly increased survival rates and ameliorated sepsis.

Only 30% of the mice who were treated with 30 mg.kg^−1^ LPS for 48 h survived. Meanwhile, the survival rate of the mice pretreated with 0.5 mg.kg^−1^ MK-801 for 48 h increased to 80% (Figure 1A). The white blood cell count of bronchoalveolar lavage fluid (BALF) in sepsis group was significantly increased compared with the control group after 24 h. Pretreatment with MK-801 can significantly inhibit the increase in white blood cell count (Table 1).

**Table 1:** Total and differential cells counts in BALF (*n*=10)

Groups	Total cells	Macrophage	Eosinophils	Neutrophils	Lymphocytes
Control	7.55 ± 0.44	6.93 ± 2.37	0.71 ± 0.27	0.21 ± 0.04	0.13 ± 0.04
LPS	15.03 ± 0.31*	11.15 ± 1.67*	0.75 ± 0.22	1.53 ± 0.08*	0.15 ± 0.05
Control + MK-801	7.44 ± 0.29	6.54 ± 1.22	0.68 ± 0.32	0.33 ± 0.08	0.12 ± 0.01
LPS + MK-801	10.87 ± 1.49^‡^	9.50 ± 1.07^†^	0.72 ± 0.63	0.62 ± 0.01^‡^	0.13 ± 0.10

Male Kunming mice were intraperitoneally administered LPS (30 mg.kg^−1^). MK-801 (0.5 mg.kg^−1^) was intraperitoneally injected before 30 min of LPS administration. The total and differential cells’ counts were analyzed in BALF. Data are presented as (mean ± S.D.) × 10^5^ cells/ml. *P<0.01, significantly different from control group. ^†^*P*<0.05.^‡^*P*<0.01 compared with significant difference from LPS group.

The results of HE staining images of lung sections showed that the bronchial and alveolar epithelial cells were clear without inflammatory cell infiltration. LPS induced infiltration of inflammatory cells in bronchial lumen, alveolar cavity, and pulmonary interstitial. MK-801 had obvious protective effects on airway inflammation and structure induced by LPS (Figure 1B), while had no effects on normal control.

### Glutamate and NMDAR expression

**Figure 2:**
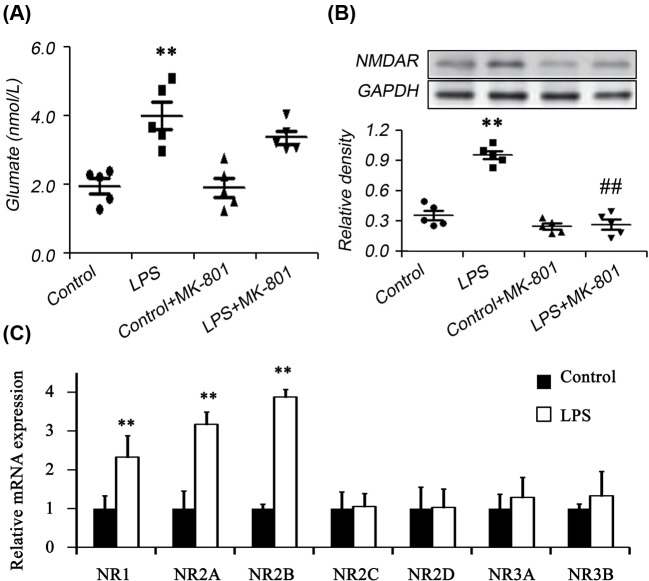
Determination of glutamate, NMDAR and NMDAR subunits Glutamate and NMDAR expression were examined by biochemical assay (**A**), Western blot (**B**), and real-time PCR (**C**) respectively (*n*=5). Male Kunming mice were intraperitoneally administered LPS (30 mg.kg^−1^). MK-801 (0.5 mg.kg^−1^) was intraperitoneally injected before 30 min of LPS administration. The secretion of glutamate in BALF was assayed by biochemical kit and the expression of NMDAR was assayed in lung tissues. The results showed that the secretion of Glutamate and the expression of NMDAR expression were enhanced in LPS-induced animal models. MK-801 could significantly reduce the expression of NMDAR in the pulmonary epithelial cells of mice. Moreover, NR1, NR2A, and NR2B increased significantly in LPS-induced animal models when compared with control. **P<0.01 compared with Control; ^##^*P*<0.01 compared with LPS group.

In order to probe the effects of possible molecular signal mechanism of glutamate signaling, the secretion of glutamate and the expression of its receptor in the lungs were analyzed. The results showed that the secretion of Glutamate was enhanced in LPS-induced animal models. Correspondingly, in the control group, the pulmonary epithelial NMDAR was rarely expressed. The intraperitoneal injection of LPS 24 h induced a large expression of NMDAR in the pulmonary epithelial cells of the mice, and the pretreatment of MK-801 could significantly reduce the expression of NMDAR in the pulmonary epithelial cells of mice (Figure 2A,B). NMDARs have seven known types of subunits (NR1, NR2 (A–D), and NR3 (A–B)). We further detected the expression levels of the seven subunits and found NR1, NR2A, and NR2B increased significantly in LPS-induced animal models when compared with control.

### MK-801 promoted VIP and inhibited SP

**Figure 3:**
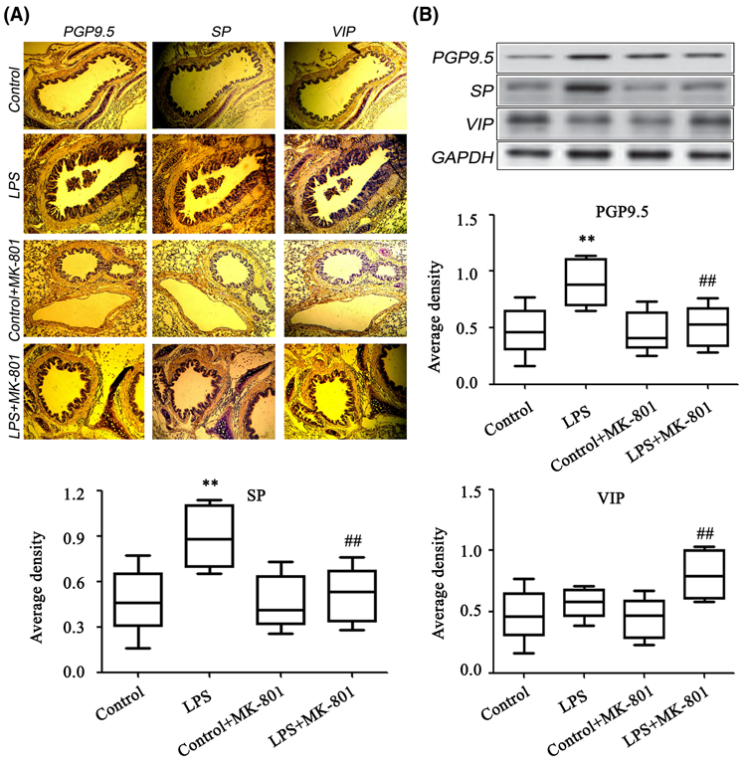
Determination of PGP9.5, SP and VIP in the lungs The expression levels of PGP9.5 SP and VIP were analyzed by immunohistochemistry (**A**) and Western blot (**B**) (*n*=5). Male Kunming mice were intraperitoneally administered LPS (30 mg.kg^−1^). MK-801 (0.5 mg.kg^−1^) was intraperitoneally injected before 30 min of LPS administration. The results from lung tissues and lung slides, showed PGP9.5 and SP increased in sepsis group and MK-801 inhibited the expression of PGP9.5 and SP. VIP was increased in MK-801-treated group. **P<0.01 compared with Control; ^##^*P*<0.01 compared with LPS group.

By using immunohistochemistry and Western blot study, we observed that the PGP9.5 increased in sepsis group and MK-801 inhibited the expression of PGP9.5 (Figure 3A). Moreover, the expression of SP was increased in the lungs in sepsis group and significantly reduced by MK-801. Additionally, the level of anti-inflammatory neuropeptide VIP was significantly increased in MK-801-treated sepsis animals. These data indicate that blockade of glutamate signaling promoted the down-regulation of SP along with the up-regulation of VIP (Figure 3B).

### NMDAR expression are related to VIP and SP release

**Figure 4:**
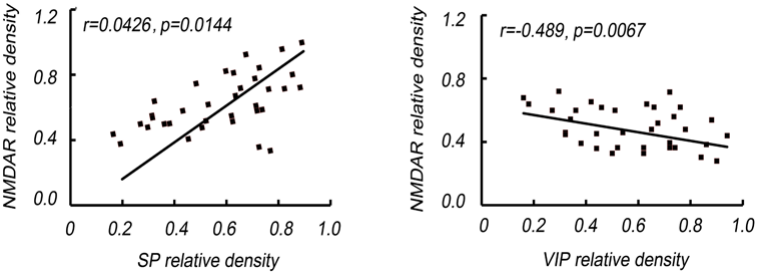
NMDAR expression is related to SP and VIP release The correlation of NMDAR expression and SP and VIP release was analyzed by simple correlation and multiple regression analyses. NMDAR expression was positively associated with SP level, while negatively correlated to VIP.

Correlated analysis results showed NMDAR expression was positively associated with SP level (*P*=0.0067), while negatively correlated to VIP (*P*=0.0144). Moreover, the relationship between NMDAR and SP or VIP level was persistent no matter the groups (Figure 4).

## Discussion

Sepsis-induced ALI is a very important and difficult clinical syndrome leading to the death of ICU patients, which has received great attention, and its pathogenesis remains unclear. ALI is characterized by accumulation of activated neutrophils, proinflammatory cytokines into the lungs as well as epithelial and endothelial dysfunction. Although early and active antibiotic therapy has been performed in patients with sepsis, this treatment is often unable to effectively remove pathogenic microorganisms. Moreover, the neurological and humoral disturbances caused by sepsis are not restored by the use of antibiotics [11]. Recent studies show that glutamate signaling may be involved in the pathogenesis of ALI.

Numerous studies have shown that glutamate and its NMDAR play an important role in cell injury. Glutamate, as an important excitatory neurotransmitter in the CNS, has been recognized and plays an important role in learning, memory, and brain development [12]. Excessive activation of NMDARs can cause Ca^2+^ overload and accumulation of Na^+^ in nerve cells. High concentration of these Ca^2+^, Na^+^ induces the activation of various signaling pathways, activation of enzyme including proteases, kinases, phosphokinases, lipoxygenases, NOS, and ROS leading to acute swelling, delayed necrosis, and apoptosis of neurones [13]. MK-801 is a noncompetitive glutamate NMDA channel blocker. Previous study showed that MK-801 decreases oxidative stress and limits inflammatory response and alveolar disarray in LPS-induced ALI [14]. Another report showed that MK-801 allievated allergic lung inflamation via endocrine outflow from the CNS to the periphery and/or as a consequence of the direct action on immune cell activity [15]. In order to probe the effects of possible molecular signal mechanism of glutamate signaling, the present study showed that the secretion of glutamate was enhanced in LPS-induced animal models. Correspondingly, in the control group, the pulmonary epithelial NMDAR was rarely expressed, and the intraperitoneal injection of LPS 24 h induced a large expression of NMDAR in the pulmonary epithelial cells of the mice, and the pretreatment of MK-801 could significantly reduce the expression of NMDAR in the pulmonary epithelial cells of mice. Moreover, MK-801 pretreatment promoted the survival rate of sepsis animal models, improved lung inflammation, indicating glutamate signaling was involved in the LPS-induced sepsis. The molecular and functional diversity of NMDARs is derived from at least seven gene family members that are alternatively spliced, leading to seven known types of subunits (NR1, NR2 (A–D), and NR3 (A–B)). The present study found NR1, NR2A, and NR2B increased significantly in LPS-induced animal models, indicating MK-801 played protective effects in ALI through NR1, NR2A, or NR2B. However, the precise mechanism needs further study.

VIP is a 28 amino acid neuropeptide, which exerts its beneficial effect against bronchial asthma via preserving normal end-expiratory lung volume, inhibiting airway inflammation, and preserving the physiological lung structure [16,17]. VIP inhibits the production of inflammatory cytokines and chemokines from macrophages, microglia, and dendritic cells. SP participates in neurogenic inflammatory response and increases the recruitment and adherence of immune cells by stimulating the expression of adhesion molecules, and increases the invasiveness of tumor by increasing adhesion between tumor cells and stromal cells [18,19]. In the initial stage of sepsis, the cascade release of inflammatory factors may result in systemic inflammatory response syndrome (SIRS) reaction and even multiple organs dysfunction syndrome (MODS). A large amount of SP release and the lack of VIP may promote the secretion of inflammatory factors in the model animal body, which aggravates the inflammatory reaction after injection of LPS, and eventually leads to the decline of early survival rate. In the present study, we found that the PGP9.5 increased in sepsis group and MK-801 inhibited the expression of PGP9.5. Moreover, the expression of SP was increased and the level of anti-inflammatory neuropeptide VIP decreased significantly in sepsis group which were reversed by MK-801. Additionally, correlated analysis showed NMDAR expression was positively associated with SP level and negatively correlated to VIP, indicating NMDAR was involved in the airway neurological modulation. The mechanism that NMDAR activation inhibited VIP and promoted SP remained unclear. However, a previous report showed that NMDA NR2B subunits containing NMDAR on small diameter primary afferents may play a role in the modulation of neurotransmitter release from primary afferent terminals, indicating that NMDAR may also play a role in neurotransmitter release from peptidergic innervations. The mechanism needs further study [20].

In conclusion, the results of the present study indicated blockade of glutamate receptors may represent a promising therapeutic strategy for the treatment of sepsis-associated ALI through neural regulation.

## Abbreviations

ALI: acute lung injury
BALF: bronchoalveolar lavage fluid
CNS: central nervous system
GAPDH: glyceraldehyde 3-phosphate dehydrogenase
HE: hematoxylin and eosin
ICU: intensive care unit
LPS: lipopolysaccharide
mGluR: metabotropic glutamate receptor
NMDA: N-methyl-d-aspartate
NMDAR: N-methyl-d-aspartate receptor
NOS: nitric oxide synthase
PGP9.5: protein gene product 9.5
ROS: reactive oxygen species
SP: substance P
VIP: vasoactive intestinal polypeptide
